# Patient weight has diverse effects on the prescribing of different antibiotics to dogs

**DOI:** 10.3389/fvets.2024.1358535

**Published:** 2024-02-19

**Authors:** Stuart D. Becker, David M. Hughes

**Affiliations:** ^1^Department of Health Data Science, Institute of Population Health, University of Liverpool, Liverpool, United Kingdom; ^2^Pathobiology and Population Sciences, The Royal Veterinary College, Hertfordshire, United Kingdom

**Keywords:** antibiotic, antimicrobial stewardship, canine, cost, treatment choice, veterinary

## Abstract

**Introduction:**

Various factors including body weight-associated treatment cost may influence the probability of dispensing antibiotics to dogs in first-opinion practice, but their effect on specific drug choice remains unclear.

**Methods:**

Multiple membership regression modeling was used to investigate the probability of dispensing 12 different antibiotics to dogs of different weights in the context of various disease presentations, using anonymized data obtained from electronic health records of 18 clinics between 2020 and 2022. Data from 14,259 dogs were analyzed.

**Results:**

Treatment choice varied significantly with animal weight. Higher body weight was associated with an increased likelihood of dispensing lower cost antimicrobials such as amoxicillin and trimethoprim sulfonamide, while use of higher cost antimicrobials such as cefovecin was strongly biased to smaller animals. However, these effects were limited when restricted treatment options were available for the target condition.

**Conclusion:**

This work demonstrates that anticipated financial costs may result in different treatment choices for canine patients depending on their body weight. Further work is needed to understand the impact of financial pressures on veterinarians’ treatment choices, and the implications for the optimization of antimicrobial stewardship in first opinion practice.

## 1 Introduction

Antimicrobials are commonly used to treat infectious diseases in veterinary and human patients. Numerous factors are known to influence the decision of veterinarians to prescribe specific antibiotics, including clinical considerations, microbiological testing, and practical aspects of treatment such as ease of dose administration and clients’ financial constraints (1, 2). Guidelines on antimicrobial use and stewardship in companion animals have been developed by several national and international animal health organizations (3, 4), but their implementation is very inconsistent among practicing veterinarians (5, 6), and high priority critically important antibiotics are used commonly in veterinary clinics worldwide (1, 7–15).

In companion animals drug dosages are calculated on the basis of body weight, typically as mg of drug per kg, although body surface area may be also used for some chemotherapeutic agents (16). Dog populations include a wide range of body weights, and when prescribing antibiotics for large dogs the quantity, and therefore the cost, of medication required can be substantially higher than for smaller animals, particularly for more expensive drugs (Table 1, Supplementary Figure S1). Financial constraints may predispose to suboptimal antibiotic treatment (19) which can manifest through unaffordability of laboratory tests (20, 21), use of inappropriately low drug doses (22), or preferential use of lower-cost medications despite lower perceived efficacy (2, 23, 24). Therefore, as treatment cost is higher in larger animals, the risk of suboptimal treatment may increase with greater patient weight.

**Table 1:** Standardized wholesale cost of treatment (in GBP)* relative to co-amoxiclav** (17).

Class	Antibiotic	Standardized cost of treatment^*^
Cephalosporins 1^st^/2^nd^ generation	Cephalexin	0.76
Cephalosporins 3^rd^/4^th^ generation	Cefovecin	2.53
Lincosamides	Clindamycin	0.63
Macrolides	Azithromycin	0.73
	Erythromycin	0.34
Nitroimidazoles	Metronidazole	1.34
Penicillins	Amoxicillin	0.15
	Co-amoxiclav	1.00
Fluoroquinolones	Enrofloxacin	0.72
	Marbofloxacin	0.81
	Pradofloxacin	0.89
Sulfonamides	Trimethoprim/sulfonamide	0.14
Tetracyclines	Doxycycline	1.67
	Oxytetracycline	0.38

*Calculated as cost per kg per day using lowest end of published dose range (16), and assuming cefovecin provides 14 days of treatment, its maximum duration of antimicrobial activity in dogs (18).

**Co-amoxiclav used here as the reference treatment as this drug was the most commonly used antibiotic in the data set.

Several previous studies have investigated veterinary antibiotic choice through surveys and questionnaires (1, 2, 7, 8, 23, 24), increasing the risk of bias or errors due to self-reporting. More objective analysis of veterinary clinical notes has been attempted using keyword analysis alongside survey data (15, 25–27), and veterinary-trained Bidirectional Encoder Representations from Transformers (BERT) natural language processing models (28), but there are few such models and further work is needed.

In this study, anonymised data were collected from first-opinion electronic health records to examine the association between animal weight and the choice of antibiotic prescribed in canine infectious disease, in the context of other animal characteristics and clinical considerations.

## 2 Materials and methods

### 2.1 Data collection

Data were obtained from 18 veterinary clinics, employing 53 vets across the period of the study, in North West England. Microsoft SQL Server 2019 (29) was used to extract information from the clinical databases, covering a period of 2 years from February 2020 to February 2022, and including only canine patients which had received antibiotic treatment within the specified time period. Antibiotics considered for inclusion in the study were amoxicillin, cefovecin, cephalexin, clindamycin, co-amoxiclav, doxycycline, enrofloxacin, erythromycin, marbofloxacin, metronidazole, oxytetracycline, pradofloxacin, and trimethoprim sulfonamide. Trimethoprim sulfonamide was not available as an authorized veterinary product for dogs at the time of this survey, so equivalent medications authorized for human use had been prescribed in cases where this antibiotic was used (30).

Records were excluded where body weight was missing, or where product cost was zero or negative (indicating a free-of-charge product replacement or refund respectively). An additional 382 animals (1.4% of records) were excluded due to incorrect recorded age (ranging from 119 to 121 years), and one animal, recorded as a Jack Russell Terrier, was excluded due to an impossibly high reported weight of 145 kg. Dog breed was recorded as free text in the data set, but as the descriptions used were extremely variable this was not deemed reliable for use as a demographic category.

### 2.2 Participant confidentiality

To ensure confidentiality and to prevent identification of veterinary clients and staff no information was collected on animal keepers, clinicians, or clinics, and the data set was provided by the source organization in anonymized form. Information collected included: date of transaction, sex, neutering status, breed, weight, age, product database code, trade name, quantity, cost, and a binary indicator showing if specific keywords were present in the concurrent clinical notes (within 24 h before or after antibiotic sale), relating to any of four major organ systems (gastrointestinal, respiratory, urinary, skin) (Supplementary material S2). Animals were allocated a unique alphanumeric code for identification.

All statistical analyses were performed using R (version 4.2.0) (31) or MLwiN (version 3.05) (32). Crude differences in antibiotic use by patient weight were examined using the Kruskal Wallis test to compare patient weight distribution between groups treated with different drugs.

### 2.3 Multiple membership modeling

**Figure 1:**
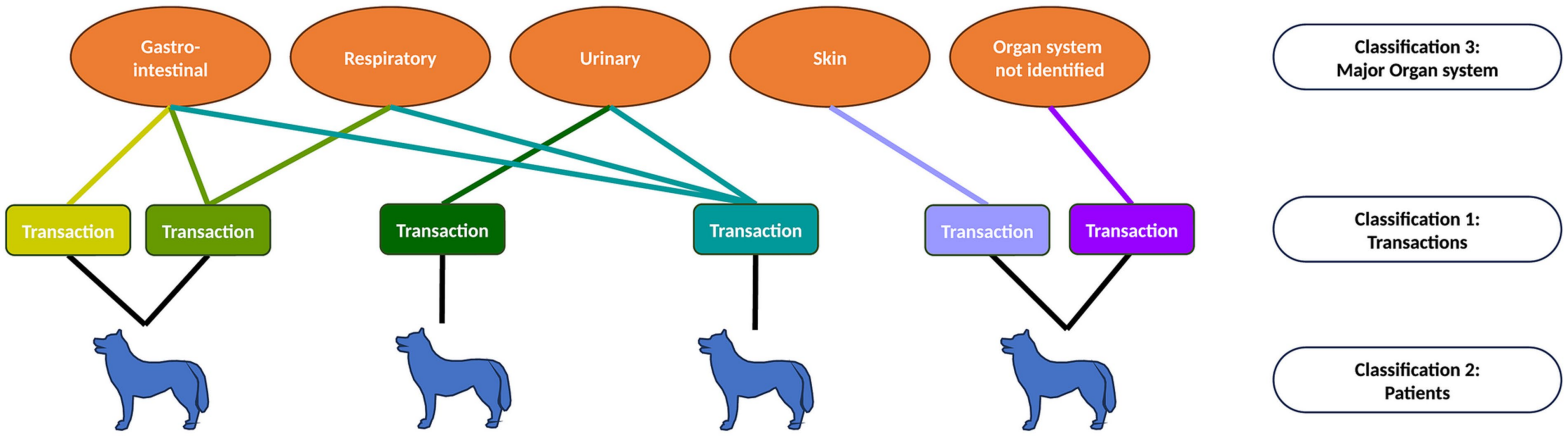
Representation of multiple membership model structure. Patients may have one or more transactions, and so transactions are nested within patients. Each transaction may be classified as belonging to one or several major organ system categories (multiple membership of ‘organ system’).

A multiple membership approach (33) was used to investigate the probability of dispensing each antibiotic in the context of patient characteristics and affected major organ systems (Figure 1). Here, multiple transactions belonging to an individual patient are likely to be more similar to each other than to transactions belonging to other animals, and thus transactions are nested within patients. In addition, each transaction may be classified under one, several or no major organ systems, meaning that transactions may be ‘members’ of multiple major organ system groups. The effect of organ system was weighted by a factor representing the reciprocal of the number of major organ systems referenced in each transaction, such that the total weights for each transaction summed to one. The model outcome variable was the log-odds of dispensing each generic antibiotic drug, and the most commonly used antibiotic (co-amoxiclav) served as the reference category. Explanatory variables included animal weight, age, sex, and neutering status, and binary indicators which flagged the presence of keywords relating to the four identified major organ systems. The effect of these additional variables on the probability of antibiotic dispensing was also assessed during analysis.

As some antibiotics have age-specific adverse effects (34) which commonly result in avoidance of these drugs for patients in discrete age categories (19) a polynomial term was included to anticipate a non-linear relationship with antibiotic dispensing probability. The square root of age was used as the preferred polynomial predictor as this obtained better model fit than age squared. Sex and neutering status were included as the prevalence of several conditions commonly treated with antibiotics in dogs varies with these factors (35, 36). Outcome was reported as odds ratios with 95% confidence intervals, and *p*-values calculated following the method described by Altman and Bland (37).

Estimates for model parameters were made in a Bayesian framework using Markov chain Monte Carlo (MCMC), with a burn-in of 10,000 iterations and an additional 3,000,000 iterations (retaining 1,000,000 iterations) to obtain model parameter estimates (38). Model convergence was assessed using effective sample size (39) calculated on individual animal-level variance (38), the Raftery-Lewis diagnostic which estimates the length of Markov chain required to estimate a particular quantile (40, 41) and visual inspection of MCMC chains. Normal and orthogonal approaches to parameterisation of MCMC models were compared using DIC, and the method obtaining best model convergence and fit was subsequently used for all MCMC parameter estimates. Diffuse gamma priors were used for variance parameters.

Due to software limitations, it was not possible to use a multiple membership approach to include major organ systems in a multinomial random effects model. Thus, a series of binomial multiple membership models separately compared the odds of dispensing each antibiotic to the reference category co-amoxiclav (Supplementary material S3). As there were no missing data, no bias in parameter estimates was anticipated as a result of this approach (42).

## 3 Results

The data set contained details of 14,259 individual animals and included 26,087 transactions where antibiotics had been dispensed, consistent with individual animals receiving multiple treatments. The mean number of antibiotic prescriptions per dog was 1.8 over the two-year period, with 60% of dogs having only one prescription, and 95% having four or fewer. The maximum was 24 prescriptions in one animal. The sex distribution was approximately equal, and just over 60% of animals were neutered (Table 2). The sex of 229 animals was recorded as ‘unknown’, and oxytetracycline was used in only nine animals. As ‘sex’ was a potentially important predictor of treatment choice, and the low number of patients dispensed oxytetracycline was unrepresentative and invalidated individual random effects, these animals were excluded from further analysis.

**Table 2:** Demographics of cases where antibiotics were dispensed*.

			Sex			Neutering status		Weight (kg)	Age (years)	Records where major organ systems were identified**				
Antibiotic (Class)	Cases	Unique patients	Female	Male	Unknown	Entire	Neutered	Mean (standard deviation)	Mean (standard deviation)	Gastro- intestinal	Respiratory	Urinary	Skin	Unspecified
Amoxicillin (Penicillin)	109	68	70 (64.2%)	39 (35.8%)	0 (0.0%)	46 (42.2%)	63 (57.8%)	25.2 (13.4)	8.8 (4.3)	18 (16.5%)	22 (20.2%)	22 (20.2%)	55 (50.5%)	44 (40.4%)
Cefovecin (3^rd^ generation cephalosporin)	151	110	76 (50.3%)	72 (47.7%)	3 (2.0%)	51 (33.8%)	100 (66.2%)	11.0 (6.1)	10.4 (4.1)	53 (35.1%)	47 (31.1%)	31 (20.5%)	95 (62.9%)	28 (18.5%)
Cephalexin (1^st^ generation cephalosporin)	1,354	1,039	598 (44.2%)	735 (54.3%)	21 (1.6%)	546 (40.3%)	808 (59.7%)	19.7 (12.4)	6.7 (4.0)	117 (8.6%)	253 (18.7%)	134 (9.9%)	1,112 (82.1%)	176 (13.0%)
Clindamycin (Lincosamide)	2,484	1,612	1,091 (43.9%)	1,360 (54.8%)	33 (1.3%)	768 (30.9%)	1716 (69.1%)	16.5 (10.5)	8.6 (4.0)	319 (12.8%)	405 (16.3%)	183 (7.4%)	1,142 (46.0%)	1,081 (43.5%)
Co-amoxiclav (Penicillin)	16,090	10,458	7,996 (49.7%)	7,852 (48.8%)	242 (1.5%)	6,325 (39.3%)	9,765 (60.7%)	18.3 (11.6)	7.0 (4.4)	3,517 (21.9%)	4,250 (26.4%)	3,570 (22.2%)	9,569 (59.5%)	3,631 (22.6%)
Doxycycline (Tetracycline)	724	503	276 (38.1%)	439 (60.6%)	9 (1.2%)	282 (39.0%)	442 (61.0%)	14.6 (9.4)	7.8 (4.5)	110 (15.2%)	375 (51.8%)	86 (11.9%)	385 (53.2%)	196 (27.1%)
Enrofloxacin (Fluoroquinolone)	465	297	214 (46.0%)	240 (51.6%)	11 (2.4%)	209 (44.9%)	256 (55.1%)	18.7 (11.1)	8.5 (4.2)	128 (27.5%)	166 (35.7%)	126 (27.1%)	255 (54.8%)	96 (20.7%)
Erythromycin (Macrolide)	183	134	85 (46.4%)	90 (49.2%)	8 (4.4%)	94 (51.4%)	89 (48.6%)	23.2 (15.7)	3.1 (3.7)	97 (53.0%)	10 (5.5%)	7 (3.8%)	40 (21.9%)	72 (39.3%)
Marbofloxacin (Fluoroquinolone)	538	318	271 (50.4%)	258 (48.0%)	9 (1.7%)	202 (37.5%)	336 (62.5%)	20.3 (12.0)	8.4 (4.1)	61 (11.3%)	94 (17.5%)	103 (19.1%)	287 (53.4%)	166 (30.9%)
Metronidazole (Nitroimidazole)	3,716	2,766	1739 (46.8%)	1917 (51.6%)	60 (1.6%)	1,382 (37.2%)	2,334 (62.8%)	18.4 (12.0)	6.4 (4.7)	3,062 (82.4%)	1,042 (28.0%)	548 (14.7%)	1,552 (41.8%)	426 (11.5%)
Oxytetracycline (Tetracycline)	68	9	10 (14.7%)	58 (85.3%)	0 (0.0%)	11 (16.2%)	57 (83.8%)	22.4 (7.0)	8.2 (3.1)	27 (39.7%)	4 (5.9%)	0 (0.0%)	7 (10.3%)	36 (52.9%)
Pradofloxacin (Fluoroquinolone)	80	55	44 (55.0%)	36 (45.0%)	0 (0.0%)	21 (26.3%)	59 (73.8%)	15.7 (8.8)	8.3 (4.3)	5 (6.3%)	14 (17.5%)	6 (7.5%)	48 (60.0%)	27 (33.8%)
Trimethoprim/sulfonamide (Sulfonamide)	125	94	52 (41.6%)	72 (57.6%)	1 (0.8%)	58 (46.4%)	67 (53.6%)	22.9 (12.2)	6.5 (4.8)	19 (15.2%)	29 (23.2%)	24 (19.2%)	66 (52.8%)	31 (24.8%)
All	26,087	14,259	12,522 (48.0%)	13,168 (50.5%)	397 (1.5%)	9,995 (38.3%)	16,092 (61.7%)	18.2 (11.6)	7.1 (4.5)	7,533 (28.9%)	6,711 (25.7%)	4,840 (18.6%)	14,613 (56.0%)	6,010 (23.0%)

*Percentages refer to cases of antibiotic dispensing. Individual animals may have more than one episode of dispensing.

**Individual animals may belong to more than one organ system category.

**Figure 2:**
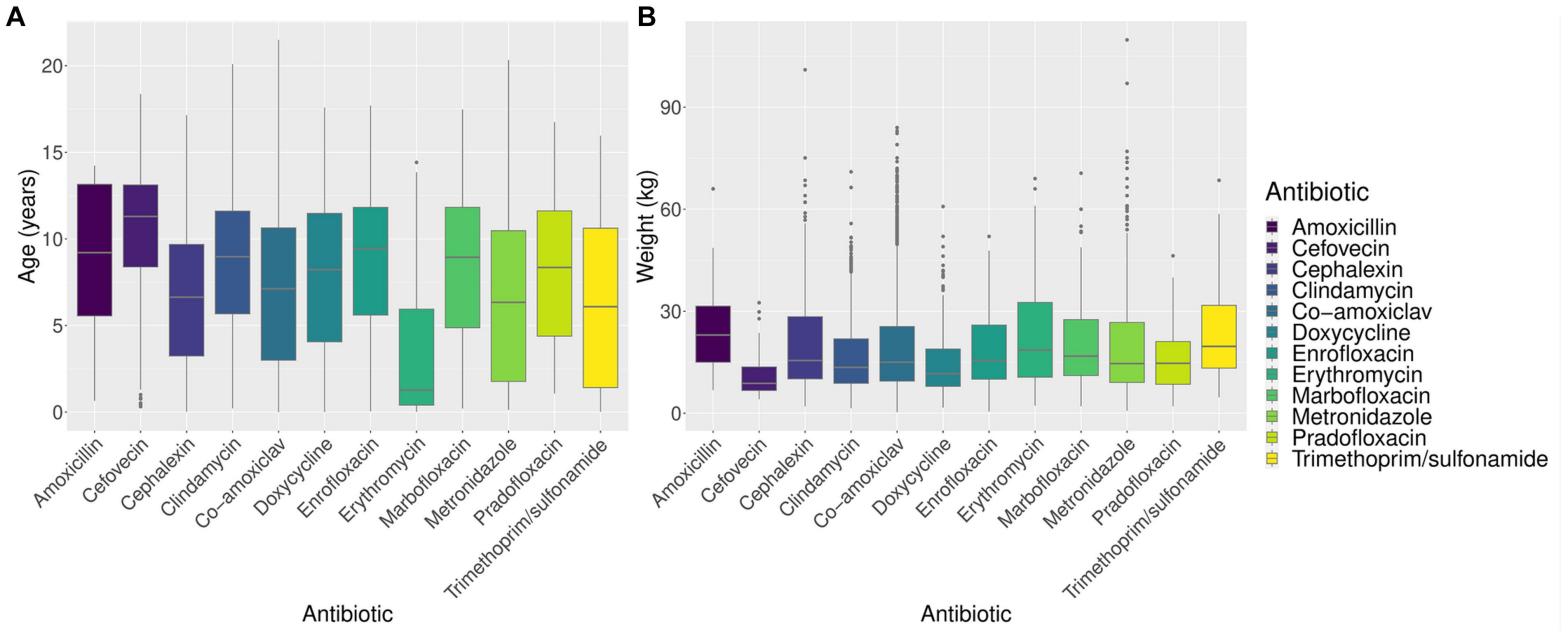
**(A)** Age distribution by antibiotic. **(B)** Weight distribution by antibiotic.

Amoxicillin use appeared higher in females. Erythromycin appeared to be used more commonly in non-neutered (entire) animals and in younger animals (Figure 2A). Cefovecin appeared to be used more commonly in animals with lower body weight (Figure 2B) and greater age, perhaps reflecting, respectively, the high relative cost of this drug (Table 1) and higher perceived health risk in these animals (19).

As found in previous studies (15), co-amoxiclav was by far the most frequent antibiotic, used in 62% of all cases, and most commonly dispensed as a 7-day treatment course. Prescriptions of other antibiotics comprised: amoxicillin 0.4% of cases; cefovecin 0.6%; cephalexin 5.2%; clindamycin 9.5%; doxycycline 2.8%; enrofloxacin 1.8%; erythromycin 0.7%; marbofloxacin 2.1%; metronidazole 14.2%; oxytetracycline 0.3%; pradofloxacin 0.3%; trimethoprim sulfonamide 0.5%. Use of different antibiotics varied significantly by body weight (*Χ*^2^ = 1937, *p* < 0.001).

References to at least one specified major organ system (gastrointestinal, respiratory, urinary, skin) were identified in clinical notes in 77% of cases. A single major organ system was identified in 42% of transactions, two organ systems In 21%, three in 10% and all four in 4%. In the remaining 23% of transactions, the target major organ system was not identified.

All predictors significantly affected the probability of dispensing a range of different antibiotics, including weight, age (linear and polynomial terms), sex, neutering status, and major organ system references. The best model fit and convergence was obtained using orthogonal parameterisation for MCMC estimation (41). Visual assessment of MCMC chains confirmed that good mixing and stationary distribution was achieved, and values for the Raftery-Lewis diagnostic indicated that the chain length was more than adequate to estimate the upper and lower 95% credible interval of all model parameters with a probability of 95%.

**Figure 3:**
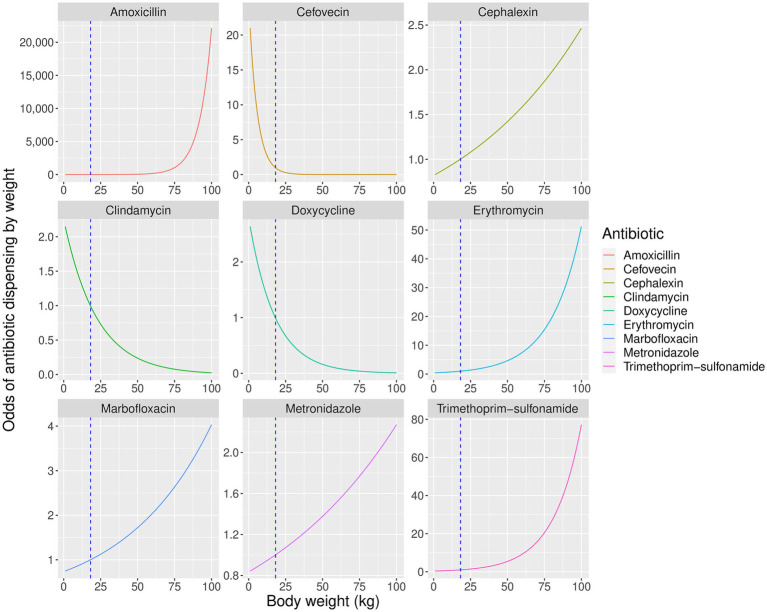
Predicted partial effects of weight on odds of dispensing significantly affected antibiotics relative to a dog of average sample weight (18 kg). Vertical dashed line represents mean population weight.

**Figure 4:**
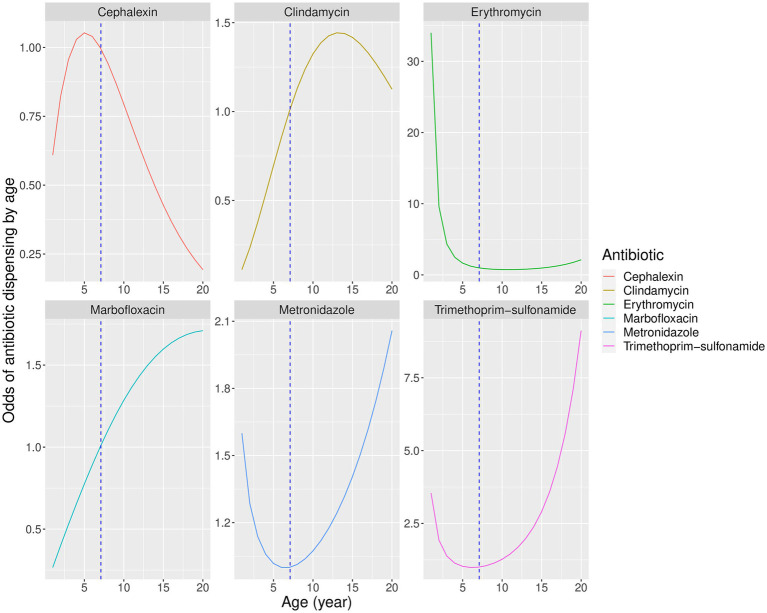
Predicted partial effects of combined age and square root of age on odds of dispensing antibiotics relative to a dog of average sample age (7 years). Vertical dashed line represents mean population age.

Body weight significantly affected the probability of dispensing all antibiotics except enrofloxacin and pradofloxacin (Table 3, Figure 3), while patient age significantly affected the dispensing of cephalexin, clindamycin, erythromycin, marbofloxacin, metronidazole and trimethoprim-sulfonamide only (Figure 4). Males were significantly more likely than females to be treated with cephalexin, clindamycin, doxycycline, and metronidazole, but no antibiotics were more commonly dispensed in females. Clindamycin and metronidazole were significantly more likely to be dispensed to neutered dogs, while enrofloxacin was more commonly used in entire animals. Metronidazole and erythromycin were significantly more likely to be dispensed when gastrointestinal references were present, doxycycline and enrofloxacin more commonly dispensed in the context of respiratory system references, and use of clindamycin was significantly reduced in urinary disorders, likely due to its lack of efficacy against several common urinary pathogens (43). There was a significant increase in use of cephalexin in the context of keywords for skin disease (Table 3).

**Table 3:** Effect of model predictors on odds ratios of dispensing each antibiotic relative to reference category co-amoxiclav.

	Amoxicillin	Cefovecin	Cephalexin	Clindamycin	Doxycycline	Enrofloxacin	Erythromycin	Marbofloxacin	Metronidazole	Pradofloxacin	Trimethoprim-sulfonamide
(Intercept)	0.00 (0.00, 0.00) *p* < 0.001	0.00 (0.00, 0.00) *p* < 0.001	0.00 (0.00, 0.00) *p* < 0.001	0.00 (0.00, 0.00) *p* < 0.001	0.00 (0.00, 0.01) *p* < 0.001	0.00 (0.00, 0.00) *p* < 0.001	0.00 (0.00, 0.22) *p* = 0.010	0.00 (0.00, 0.00) *p* < 0.001	0.12 (0.01, 2.58) *p* = 0.176	0.00 (0.00, 0.00) *p* < 0.001	0.00 (0.00, 0.00) *p* < 0.001
Weight (kg)	1.13 (1.06, 1.21) *p* < 0.001	0.84 (0.78, 0.89) *p* < 0.001	1.01 (1.00, 1.02) *p* = 0.028	0.96 (0.95, 0.97) *p* < 0.001	0.94 (0.93, 0.96) *p* < 0.001	1.00 (0.99, 1.02) *p* = 0.908	1.05 (1.02, 1.08) *p* = 0.001	1.02 (1.00, 1.03) *p* = 0.015	1.01 (1.00, 1.02) *p* = 0.001	0.96 (0.92, 1.01) *p* = 0.139	1.05 (1.02, 1.09) *p* = 0.003
Age (year)	0.69 (0.31, 1.53) *p* = 0.370	1.34 (0.89, 2.02) *p* = 0.157	0.71 (0.62, 0.81) *p* < 0.001	0.69 (0.61, 0.79) *p* < 0.001	0.96 (0.81, 1.15) *p* = 0.702	1.02 (0.82, 1.26) *p* = 0.899	2.09 (1.40, 3.11) *p* < 0.001	0.86 (0.69, 1.08) *p* = 0.191	1.22 (1.13, 1.31) *p* < 0.001	0.68 (0.35, 1.33) *p* = 0.266	1.77 (1.13, 2.78) *p* = 0.013
Sex (male)	0.47 (0.13, 1.67) *p* = 0.245	1.04 (0.51, 2.11) *p* = 0.925	1.44 (1.16, 1.79) *p* = 0.001	1.75 (1.43, 2.15) *p* < 0.001	2.13 (1.54, 2.95) *p* < 0.001	1.19 (0.82, 1.72) *p* = 0.372	0.95 (0.49, 1.86) *p* = 0.898	0.96 (0.68, 1.36) *p* = 0.841	1.35 (1.17, 1.55) *p* < 0.001	0.96 (0.35, 2.62) *p* = 0.940	1.77 (0.76, 4.11) *p* = 0.186
Neutered	0.37 (0.10, 1.37) *p* = 0.138	1.43 (0.67, 3.07) *p* = 0.366	0.85 (0.68, 1.07) *p* = 0.165	1.45 (1.17, 1.81) *p* = 0.001	1.07 (0.77, 1.50) *p* = 0.685	0.52 (0.36, 0.77) p = 0.001	1.27 (0.63, 2.57) *p* = 0.521	1.02 (0.71, 1.46) *p* = 0.925	1.54 (1.33, 1.78) *p* < 0.001	2.18 (0.71, 6.75) *p* = 0.177	0.96 (0.41, 2.27) *p* = 0.938
Square root of age ( $\sqrt{\mathrm{year}}$ )	11.85 (0.25, 567.39) *p* = 0.212	1.00 (0.13, 7.64) *p* = 0.998	4.77 (2.58, 8.83) *p* < 0.001	14.44 (7.49, 27.84) *p* < 0.001	1.44 (0.63, 3.27) *p* = 0.391	1.72 (0.62, 4.77) *p* = 0.300	0.01 (0.00, 0.05) *p* < 0.001	3.82 (1.29, 11.29) *p* = 0.015	0.37 (0.26, 0.51) *p* < 0.001	12.22 (0.42, 356.44) *p* = 0.146	0.06 (0.01, 0.44) *p* = 0.006
Gastro-intestinal	0.91 (0.30, 2.73) *p* = 0.875	2.42 (0.56, 10.48) *p* = 0.239	0.26 (0.05, 1.21) *p* = 0.085	1.19 (0.29, 4.89) *p* = 0.819	0.33 (0.04, 2.67) *p* = 0.304	1.12 (0.47, 2.68) *p* = 0.810	194.42 (1.63, 23211.43) *p* = 0.031	0.59 (0.27, 1.27) *p* = 0.177	79.20 (3.66, 1711.76) *p* = 0.005	0.26 (0.02, 3.37) *p* = 0.306	0.94 (0.57, 1.55) *p* = 0.823
Respiratory	0.62 (0.15, 2.55) *p* = 0.522	0.99 (0.30, 3.25) *p* = 0.990	1.06 (0.23, 4.84) *p* = 0.945	0.82 (0.20, 3.33) *p* = 0.789	12.72 (1.66, 97.65) *p* = 0.014	2.60 (1.09, 6.22) *p* = 0.031	0.02 (0.00, 2.56) *p* = 0.111	0.95 (0.49, 1.86) *p* = 0.895	0.73 (0.03, 15.69) *p* = 0.849	0.97 (0.11, 8.67) *p* = 0.981	1.10 (0.66, 1.84) *p* = 0.733
Urinary	1.25 (0.38, 4.18) *p* = 0.728	0.42 (0.10, 1.84) *p* = 0.251	0.60 (0.13, 2.78) *p* = 0.529	0.17 (0.04, 0.69) *p* = 0.013	0.22 (0.03, 1.74) *p* = 0.150	0.87 (0.36, 2.06) *p* = 0.758	0.05 (0.00, 7.72) *p* = 0.250	0.96 (0.50, 1.85) *p* = 0.914	0.13 (0.01, 2.75) *p* = 0.189	0.36 (0.04, 3.63) *p* = 0.396	1.00 (0.63, 1.60) *p* = 0.988
Skin	0.98 (0.37, 2.59) *p* = 0.975	1.08 (0.37, 3.19) *p* = 0.898	7.08 (1.59, 31.46) *p* = 0.010	1.52 (0.38, 6.09) *p* = 0.568	0.71 (0.09, 5.40) *p* = 0.756	0.56 (0.25, 1.26) *p* = 0.160	0.68 (0.01, 75.93) *p* = 0.882	1.08 (0.59, 1.97) *p* = 0.808	0.20 (0.01, 4.38) *p* = 0.314	3.43 (0.43, 27.62) *p* = 0.249	0.94 (0.61, 1.43) *p* = 0.771
Organ system unspecified	1.43 (0.50, 4.11) *p* = 0.518	0.92 (0.32, 2.68) *p* = 0.893	1.42 (0.32, 6.30) *p* = 0.659	4.33 (1.08, 17.35) *p* = 0.038	1.78 (0.24, 13.46) *p* = 0.586	0.71 (0.32, 1.57) *p* = 0.402	10.36 (0.09, 1139.20) *p* = 0.335	1.73 (0.94, 3.17) *p* = 0.077	0.46 (0.02, 9.90) *p* = 0.633	3.17 (0.41, 24.49) *p* = 0.272	1.03 (0.69, 1.55) *p* = 0.883

Significant coefficients are highlighted.

## 4 Discussion

As anticipated, animal body weight significantly affected the probability of prescribing most of the antibiotics in this study. The relationship between weight and odds of dispensing was non-linear (Figure 3), a pattern that was particularly pronounced for amoxicillin and cefovecin which were used almost exclusively in the heaviest and lightest patients, respectively. Overall, the impact of body weight on antibiotic choice appears complex. Where several suitable treatments exist for a particular condition, clinicians may avoid expensive antibiotics or choose a similar but less efficacious alternative in larger animals to reduce costs. However, where treatment options for the target condition are restricted, or cost is not prohibitive, the consideration of body weight may take lower priority than other factors such as specific clinical need, perceived safety profile, or dosing convenience.

### 4.1 The association with weight

Amoxicillin was more likely to be dispensed to heavier animals, suggesting its lower cost (Table 1) increased its use in animals that required larger doses of medication, despite its lack of efficacy in treating beta-lactamase positive organisms (44). Conversely, more expensive cefovecin was used almost exclusively in animals of low body weight. Cefovecin is available only as an injectable medication, where the product data sheet states that a single injection provides up to 14 days of continuous antibiotic treatment in dogs (18, 45). Its use only in smaller animals suggests this was not driven by clinical necessity, but instead may have been a convenient approach to dosing or improving treatment compliance where the cost of treatment was not prohibitive.

The probability of dispensing cephalexin was increased in larger animals, a pattern which coincided with a decrease in the use of co-amoxiclav in animals of similar weight. Both cephalexin and co-amoxiclav show efficacy against beta-lactamase-positive organisms not susceptible to amoxicillin (46). Clinicians may have replaced co-amoxiclav with lower-cost cephalexin in larger animals to retain a similar antibacterial spectrum at lower cost, despite cephalexin’s lower effectiveness (46).

Clindamycin was more likely to be dispensed in smaller animals, perhaps reflecting its utility in dental disease (16) which is more common in small-breed dogs (47). Also, in smaller animals doxycycline was more frequent, and while both doxycycline and co-amoxiclav are advised for treatment of canine respiratory disease (48), the higher price of the former would be more apparent in larger patients.

The probability of prescribing erythromycin and metronidazole was higher in larger animals, suggesting preferential use for gastrointestinal disease in these patients where co-amoxiclav could have been considered as a possible alternative (16). As metronidazole was more expensive than co-amoxiclav, routine clinical use of this antibiotic for canine diarrhea (49) appeared to override cost concerns in this instance.

Marbofloxacin and trimethoprim-sulfonamide were also more likely to be used in larger animals. The substantially lower cost of trimethoprim-sulfonamide compared to co-amoxiclav may explain its use here. However, this is not the case for marbofloxacin which is a more costly fluroquinolone than enrofloxacin. Preferential use of marbofloxacin in larger animals may reflect the rarity of reported iatrogenic cartilage damage in large-breed dogs treated with this drug compared to others in the same class (16, 50).

### 4.2 The association with age

Analysis confirmed the anticipated non-linear relationship between age and antibiotic dispensing, and suggested that for some antibiotics, higher and lower probabilities of dispensing tended to occur within distinct age ranges (Figure 4). Cephalexin use was higher in younger animals, suggesting that co-amoxiclav was preferred in older patients perhaps due to its similar antibacterial spectrum and greater effectiveness (46). Clindamycin increased in older animals, which the authors speculate may be due to use in periodontal disease (51) which occurs more commonly in older patients (16, 52).

Marbofloxacin also tended to be used in older patients, perhaps due to clinician perception of high potency with low toxicity (53), the convenience of once-daily dosing (16), or concerns about antimicrobial resistance with alternative options (54). Erythromycin was used almost exclusively in young animals, suggesting association with juvenile *Campylobacter* enteritis (16, 55, 56).

Metronidazole and trimethoprim-sulfonamide showed a bimodal distribution. Metronidazole is commonly used to treat suspected *Giardia* enteritis in the younger age group (57, 58), and trimethoprim-sulfonamide is effective against coccidiosis, also common in juvenile dogs (16, 59). In older animals metronidazole may be used to treat chronic enteropathy or inflammatory bowel disease (60), but trimethoprim-sulfonamide is effective against a broad range of infections and the reason for its increased use in older animals in this study is unclear.

### 4.3 The association with sex and neutering status

Male dogs show significantly higher prevalence of several diseases that may require antibiotic treatment, including traumatic injury, moist dermatitis, upper respiratory tract disorders, and foreign bodies which may cause peritonitis or infected wounds (35, 61). The antibiotics observed to be more commonly used in males in this study would be appropriate choices for this range of conditions (16) (Table 3).

Increased use of clindamycin in neutered animals is consistent with higher prevalence of periodontal disease in this group (35, 51), while a reported increased risk of inflammatory bowel disease in these patients (36) may explain use of metronidazole, although evidence of higher enteropathy risk is not a consistent finding (35). The risk of reproductive disorders is higher in entire animals (35), and it is possible that antibiotic treatment for these conditions may have contributed to the higher use of enrofloxacin in these patients (48, 62). While enrofloxacin is advised as appropriate treatment for canine prostatitis (48), around 50% of entire animals dispensed enrofloxacin in this study were female, suggesting cases of prostatitis only partially explained its use.

### 4.4 The association with keywords identifying major organ systems

Significant changes to antibiotic use associated with major organ system keywords generally corresponded to clinical guidelines relating to conditions in the relevant organ system. Thus, use of erythromycin and metronidazole was significantly increased with gastrointestinal keywords (55, 58), doxycycline and enrofloxacin with respiratory disease (48), and cephalexin with skin conditions (48), while clindamycin was significantly avoided with urinary disease where it is unlikely to offer effective treatment (43) (Table 3).

There was some evidence of underutilisation of condition-specific treatment options recommended in stewardship guidelines. For example, combinations of fluoroquinolones such as enrofloxacin with co-amoxiclav or clindamycin have been suggested as suitable treatment for pneumonia (48). Post-hoc analysis suggested that combinations of enrofloxacin with co-amoxiclav were sometimes used but this was not the case for clindamycin (Supplementary Table S4). Recommendations for treatment of urinary or skin disease with trimethoprim sulfonamide (48) also appear to have been underused.

While four major organ systems were chosen as common targets for antibiotic treatment for the purposes of this study (25), it is recognized that this practical adjustment was incomplete and meant that many conditions affected major organ systems not identified by these categories. Clindamycin was significantly more likely to be dispensed when no specific keywords were identified, perhaps due to its common usage in periodontal disease (52), which was not targeted for keyword identification in this study.

### 4.5 Link to previous findings

Preferential use of costly, high-priority critically important antimicrobials in animals of low weight has been demonstrated previously (63). This study confirms this finding and additionally demonstrates that larger animals are more likely to receive certain lower cost drugs. A decrease in antimicrobial dispensing probability in dogs has been reported in older (15) and neutered animals (27), but here we demonstrate this is inconsistent and varies between different antibiotics. Interestingly, previous studies have not identified strong effects of sex on antimicrobial dispensing (27, 63), suggesting that higher use in males may be masked unless differences between specific drugs are taken into account.

Clinical signs are widely recognized as important determinants of antimicrobial choice (1, 2, 19), and thus the significance of major organ system category here is unsurprising. Identification of high-priority conditions with potential for optimisation of antibiotic treatment is a key strategy for antimicrobial stewardship interventions in human medicine (64), and evidence in this study supports adoption of a similar approach in veterinary practice.

### 4.6 Implications for optimizing antimicrobial stewardship

Results from this study suggest that in some circumstances veterinarians may change their choice of antibiotic to reduce anticipated treatment cost, and this may not always conform to published guidelines. This observation appears consistent with previous studies that have demonstrated how the inhibiting effect of cost on use of culture and susceptibility testing can undermine optimal antimicrobial stewardship practices, resulting in cases of antibiotic prescribing that may not be justified to recommended standards (21, 65).

Several potential drivers of clinician decision-making identified in earlier studies may have influenced the perceived suitability of antibiotics prescribed here. These include precautionary treatment motivated by clinicians’ concerns that failure to correctly diagnose and treat an infection might compromise patient welfare (21, 65), perceived and actual social pressure, including use of guilt by some animal owners to reduce the cost of treatment (65–67), and fear that clients would be lost to competitors in the case of treatment failure if antibiotics were not provided (65).

Unlike in the UK where this study was undertaken, regulations in several other European nations have introduced mandatory culture and susceptibility testing before prescribing the highest-priority critically important antibiotics such as fluoroquinolones and third generation cephalosporins. This has been associated with substantial reductions in the use of these drugs by veterinarians in those countries (68). Veterinary clinicians value clear antimicrobial stewardship policies (21), and it is interesting to speculate that mandating some stewardship practices through regulation may also assist clinicians in the UK in following best-practice guidelines when discussing a prudent approach to antibiotic use with animal owners.

### 4.7 Limitations

As some contributing surgeries were staffed mainly by one clinician, identification codes for individual vets and clinics were excluded from the data set, to avoid the risk that confidentiality might be compromised through pattern recognition of cases associated with specific clinics or dates. While there is a theoretical risk that unidentified individual clinicians exhibiting extreme overuse of specific antibiotics may bias the results, the authors consider this to be very unlikely. All surgeries included in the study were owned by the same veterinary group, the data set included contributions from 53 clinicians, and many vets employed by this group work in more than one surgery. As a result, the approach to antibiotic usage is likely to be similar between surgeries, and unusual prescribing behavior would be noted and questioned by colleagues.

### 4.8 Conclusion

This study has demonstrated that weight and treatment cost affect veterinarians’ choice of antibiotics in ways that are not consistent between different drugs, and independent of the influence of other patient characteristics and clinical considerations. This suggests that further work is needed to understand how financial pressures may influence veterinarians’ antimicrobial treatment decisions, and the impact this may have on potentially undermining optimization of antimicrobial stewardship in companion animal veterinary practice.

## Data availability statement

The datasets presented in this article are not readily available because the data used for this study are not publicly available due to privacy or ethical restrictions. Requests to access the datasets should be directed to SB, sbecker@rvc.ac.uk.

## Author contributions

SB: Writing – original draft, Writing – review & editing. DH: Writing – review & editing.
