# Supplementary material for: Patient weight has diverse effects on the prescribing of different antibiotics to dogs
Source: Front Vet Sci. 2024 Feb 19;11:1358535. doi: 10.3389/fvets.2024.1358535 (PMC10910008; doi:10.3389/fvets.2024.1358535)
Supplement: Supplementary file 1 [file Data_Sheet_1.pdf]

Supplement to: Patient weight has diverse effects on the prescribing of different antibiotics to dogs

Stuart D. Becker, David M. Hughes

Supplementary Figure S1 – Relationship between canine patient weight and cost of treatment course.

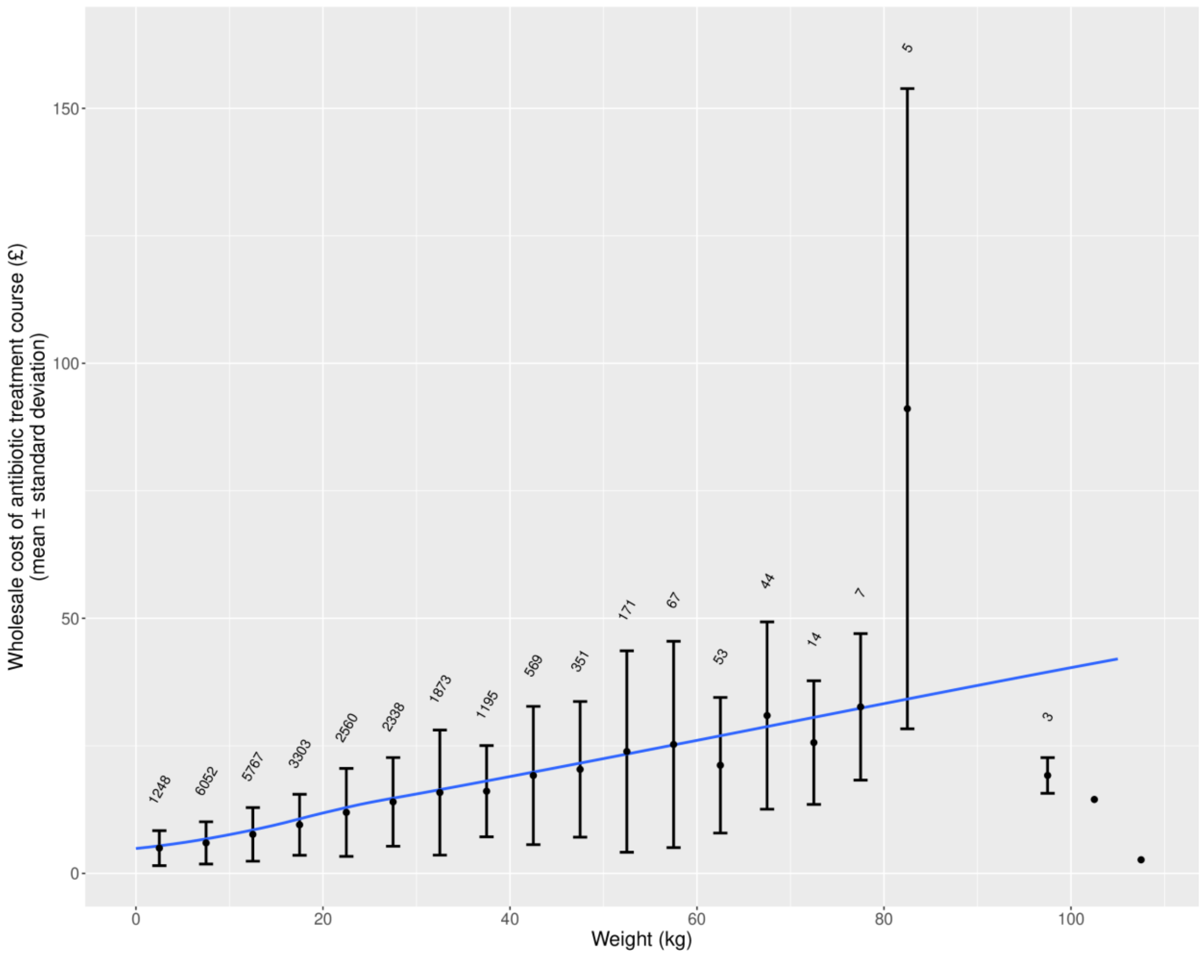

Figures above error bars represent number of patients in each 5kg weight group. Spearman's $\rho = 0.571$, $p < 0.001$.

Supplementary Material S2 – Keywords used to identify major organ systems.

Major organ systems were chosen on the basis that they had been identified previously as common targets of antibacterial therapy[1], and also that there were products used uniquely in that organ system whose descriptions could be used to create a keyword list to search for relevant clinical notes. Initially all canine clinical notes in the databases within the two-year time period were searched for a single keyword likely to be unique to clinical disease affecting each specific organ system. Keywords used were: 'probiotic' (for the gastrointestinal system), 'Corvental' (a trade name of the drug theophylline, licenced for respiratory compromise), 'Cystaid' (the trade name of a nutraceutical used in urinary disease), and 'Isaderm' (the trade name of a topical steroid and antibiotic cream used for focal skin lesions)[2-4]. All individual words from clinical notes that had been so identified were pooled and collated into a frequency table. The 1000 most frequently used words in the context of each major organ system were assessed manually, and those specifically relating to the major organ system of interest were retained. The resulting organ system-specific keyword lists were then used to search all clinical records where antibiotics had been dispensed in dogs, producing the binary indicator showing whether or not each major organ system had been referenced in clinical notes.

References

1. Singleton DA, Rayner A, Brant B, et al. A randomised controlled trial to reduce highest priority critically important antimicrobial prescription in companion animals. *Nat Commun*, 2021. **12**(1593). https://doi.org/10.1038/s41467-021-21864-3.
2. Veterinary Medicines Directorate. 2022. Product Information Database. Available: https://www.vmd.defra.gov.uk/productinformationdatabase/ [Accessed 1 July 2022].
3. Jensen A, Bjornvad C. Clinical effect of probiotics in prevention or treatment of gastrointestinal disease in dogs: A systematic review. *J Vet Intern Med*, 2019. **33**(5):1849-1864. https://doi.org/10.1111/jvim.15554.
4. VetPlus. Cystaid. 2021 [Accessed 16 Sept 2022]. Available from: https://www.vetplus.co.uk/products/cystaid/.

Gastrointestinal	Respiratory	Urinary		Skin	
bile	airway	bladder	urinating	alopecia	malaseb
colitis	breathing	crystals	urination	apoquel	mites
cpli	bronchitis	cystaid	urine	atopic	nails
d+	chest	cystitis	urolithiasis	axilla	otitis
diarrhoea	corvental	cystotomy	uti	axillae	papules
f+	cough	dipstick	uti.	bathing	paws
faecal	coughed	dysuria		chin	pinna
faeces	coughing	haematuria		collarettes	pinnae
intestines	coughs	incurin		crusting	pododermatitis
omeprazole	hacking	pollakiuria		crusts	pruritic
pancreatitis	harsh	pollakuria		crusty	pruritis
prevomax	honking	propalin		demodex	pruritus
probiotic	kc	prostate		dermatitis	pustular
probiotics	laryngeal	prostatic		digits	pustules
promax	larynx	proteinuria		dorsum	pyoderma
rectal	lung	pupd		ear	scab
stomach	lungs	renal		ears	scabby
stool	nasal	sediment		epidermal	scrape
stools	phlegm	sedivue		erythema	scrapes
v+	pulmonary	sg		erythematous	scrotal
vomit	resp	sg.		exudative	scrotum
vomited	respiratory	sg=		flea	shampoo
vomiting	sneeze	stranguria		groin	skin
	sneezing	struvite		hair	toes
	thorax	urin		hotspot	ventrum
	throat	urinalysis		interdigital	yeasty
	trachea	urinary		isaderm	
	tracheal	urinate		itchy	
	wheezy	urinated		lip	

Supplementary Material S3: Form of multiple membership random effects model for antibiotic dispensing (outcome as log-odds ratio)

$$log\left(\frac{\pi_{ij}}{1-\pi_{ij}}\right) = \beta_0 + \beta_1 Weight_j + \beta_2 Age_j + \beta_3 Sex_j + \beta_4 Neutered_j + \beta_5\sqrt{Age_j}$$
$$+ \Sigma_{k\in \mathrm{OrganSystem(i)}} w_{k,i}^{(3)} u_k^{(3)} + u_{0,Animal\_ID(i)}^{(2)} \qquad k = 1, \ldots, 5$$
$$\left[u_{OrganSystem(i)}^{(3)}\right] \sim N\left(0, \Omega_u^{(3)}\right)$$
$$\left[u_{Animal\_ID(i)}^{(2)}\right] \sim N\left(0, \Omega_u^{(2)}\right)$$

Where: Superscript numbers denote random effects of animal identifier (2) and major organ system (3).

Outcome is the log-odds of dispensing the specific antibiotic of interest in transaction *i*, nested within animal identifier *j*

$w_{k,i}^{(3)}$ measures the proportion of the transaction *i* allocated to major organ system *k*, with associated effect $u_k^{(3)}$.

Coded values for major organ system *k* are: 1 Gastrointestinal, 2 Respiratory, 3 Urinary, 4 Skin, 5 No major organ system identified.

Supplementary Table S4: Number of transactions where two antibiotics were dispensed simultaneously, for combined treatment in the same animal.

	Amoxicillin	Cefovecin	Cephalexin	Clindamycin	Co-amoxiclav	Doxycycline	Enrofloxacin	Erythromycin	Marbofloxacin	Metronidazole	Pradofloxacin	Trimethoprim-sulfonamide
**Amoxicillin**		0	0	0	1	0	1	0	0	1	0	0
**Cefovecin**	0		0	0	1	0	0	0	0	0	0	0
**Cephalexin**	0	0		3	10	1	0	0	3	6	0	0
**Clindamycin**	0	0	3		71	0	2	0	2	2	0	0
**Co-amoxiclav**	1	1	10	71		4	58	2	83	189	14	1
**Doxycycline**	0	0	1	0	4		0	0	1	1	0	0
**Enrofloxacin**	1	0	0	2	58	0		0	1	15	0	1
**Erythromycin**	0	0	0	0	2	0	0		0	1	0	1
**Marbofloxacin**	0	0	3	2	83	1	1	0		10	0	0
**Metronidazole**	1	0	6	2	189	1	15	1	10		1	0
**Pradofloxacin**	0	0	0	0	14	0	0	0	0	1		0
**Trimethoprim-sulfonamide**	0	0	0	0	1	0	1	1	0	0	0	
